# Association between subchronic and chronic lead exposure and levels of antioxidants and chemokines

**DOI:** 10.1007/s00420-016-1144-4

**Published:** 2016-06-13

**Authors:** Michał Dobrakowski, Aleksandra Kasperczyk, Natalia Pawlas, Ewa Birkner, Edyta Hudziec, Ewa Chwalińska, Sławomir Kasperczyk

**Affiliations:** 1 Department of Biochemistry, School of Medicine with the Division of Dentistry, Medical University of Silesia, ul. Jordana 19, 41-808 Zabrze, Poland; 2 Institute of Occupational Medicine and Environmental Health in Sosnowiec, ul. Kościelna 13, 41-200 Sosnowiec, Poland

**Keywords:** Subchronic exposure to lead, Chronic exposure to lead, Non-enzymatic antioxidants, Uric acid, Chemokines

## Abstract

**Purpose:**

This study aimed to compare the influence of lead on the non-enzymatic antioxidant defenses and the levels of chemokines in workers subchronically and chronically exposed to lead.

**Methods:**

The study population was divided into three groups. The first group consisted of male workers subchronically exposed to lead for 40 ± 3.2 days, while the second group included male workers chronically exposed to lead. The third group was a control group.

**Results:**

The levels of uric acid and bilirubin were significantly higher after a subchronic exposure to lead compared to the baseline by 22 and 35 %, respectively. Similarly, the values of total antioxidant capacity (TAC), total oxidant status (TOS), and oxidative stress index (OSI) increased by 15, 50, and 33 %, respectively. At the same time, the levels of thiol groups and albumin decreased by 5 and 8 %, respectively. Additionally, the levels of interleukin-8 (IL-8) and macrophage inflammatory protein-1β (MIP-1β) were significantly higher after a subchronic exposure to lead compared to the baseline by 34 and 20 %, respectively. Moreover, IL-8 level was significantly higher by 40 % in the group of workers chronically exposed to lead than in the control group, while the level of interferon gamma-induced protein-10 (IP-10) was significantly lower by 28 %.

**Conclusions:**

Similar to chronic lead exposure, subchronic exposure to lead is associated with elevated blood levels of uric acid and bilirubin in humans. This probably results in increased TAC value despite thiol depletion. However, the compensatory activation of non-enzymatic antioxidant defenses seems to be insufficient to protect against lead-induced oxidative stress, which may be additively enhanced by the pro-inflammatory action of chemokines, especially IL-8.

## Introduction

Lead is a heavy metal, which has been widely used for decades in paint, gasoline, water pipes, storage batteries, and many other products (Lin et al. 2015). In the last decades, our view on lead toxicity has changed, giving more concern to exposures, previously considered safe. Lead can induce many adverse health effects on various body systems including the nervous, hematological, immune, and genitourinary systems. Nevertheless, due to its malleability, resistance to corrosion, and low melting point, lead is still widely used in many industries (Wang et al. 2012).

Ingestion and inhalation are the primary routes of lead entering the body. After absorption, lead is distributed through the bloodstream to various organs, such as brain, liver, and kidneys (Alya et al. 2015). It has been proposed that kidneys play an important role in the toxicokinetics of lead because they serve as the major organ responsible for its excretion. Therefore, kidneys are particularly exposed to lead toxicity. Lead primarily impairs the function and structure of the renal tubules. Renal tubular epithelial cell necrosis, leukocyte infiltration, and tubular epithelial cell pyknosis have been shown to be induced by lead toxicity. Many studies indicated that long‐term exposure to lead increases the risk of nephropathy, which manifests as increased levels of renal dysfunction biomarkers, such as plasma creatinine and uric acid (Alya et al. 2015; Liu et al. 2012).

Several mechanisms have been proposed to explain lead-induced toxicity. One of them implicated oxidative stress as the underlying mechanism of toxicity (Wang et al. 2012). Oxidative stress results from imbalance between the generation and utilization of reactive oxygen species (ROS). Lead ions have been shown to be associated with increased generation of ROS. Besides, lead is able to dysregulate the antioxidant defenses, including the antioxidant enzymes and the non-enzymatic antioxidants, such as uric acid. (Dobrakowski et al. 2014; Soliman et al. 2015; Wang et al. 2012). In our previous study, we reported higher levels of uric acid in workers chronically exposed to lead compounds (Blood lead (PbB) = 40.40 ± 10.05 µg/dl) for, on average, 16.40 ± 10.20 years compared to the unexposed control group (PbB = 6.39 ± 2.47 µg/dl). Besides, we reported increased levels of other non-enzymatic antioxidants, such as bilirubin, albumin, thiol groups, and α-tocopherol (Dobrakowski et al. 2014). However, the possible association between lead toxicity and the non-enzymatic antioxidant defenses is not fully understood and needs further investigation.

Negative effects of lead on human health may be also due to its impact on the immune system function. It has been shown that lead impairs the function of lymphocytes and cytokine production. Some studies indicate lead-induced shift in the balance of T-helper (Th) lymphocyte function toward Th2-mediated immune response at the expense of Th1-mediated response (García-Lestón et al. 2012; Hsiao et al. 2011). Besides, lead exerts pro-inflammatory properties. In experimental and human studies, lead exposure has been shown to induce expression of pro-inflammatory cytokines, such as tumor necrosis factor-α (TNF-α), interleukin-6 (IL-6), and interleukin-1β (IL-1β) via activation of mitogen-activated protein kinases (MAPKs) and nuclear factor kappa B (NF-κB) (Liu et al. 2012). Both MAPKs and NF-κB can be induced synergistically by uric acid (Kanellis et al. 2003; Liang et al. 2015). Moreover, uric acid exerts its pro-inflammatory action via increasing the levels of chemokines, such as interleukin-8 (IL-8), monocyte chemoattractant protein-1 (MCP-1), and RANTES (regulated on activation, normal T cell expressed and secreted) (Liang et al. 2015; Zhou et al. 2012). Similarly, lead has been shown to induce interleukin-8 (IL-8), a potent chemokine (Lin et al. 2015; Yang et al. 2014).

Up-to-date studies indicate lead-induced dysregulation of the immune response in humans. However, in the available literature, there are no conclusive epidemiological studies regarding the immunotoxic effects of lead in occupationally exposed workers. Similarly to mechanisms underlying the associations between lead and the non-enzymatic antioxidant defense, the exact mechanisms of lead interactions with the immune system in humans still remain unclear (García-Lestón et al. 2012). Some authors postulate that the influence of lead on the immune response is dose-dependent (Hsiao et al. 2011; Iavicoli et al. 2006). Duration of exposure seems to be the second factor modifying interactions of lead with the immune system. In light of this, we decided to examine workers subchronically and chronically exposed to lead. We focused on the influence of lead on the non-enzymatic antioxidant defenses, including uric acid, and chemokine levels, which may be related to the immunomodulatory properties of both lead and uric acid.

## Materials and methods

### Study population

Each study subject provided a written consent to a study. Questionnaire data on age, weight, height, medical history, and smoking were obtained. The levels of lead and zinc protoporphyrin in the blood served as biomarkers of lead exposure.

The study population was divided into three groups. The first group consisted of 34 male workers subchronically exposed to lead for 36–44 days, aged from 22 to 61 years. Subjects in this group worked in lead–zinc works to perform periodic maintenance of blast furnaces and production lines. Among the exposed population, 17 workers were occupationally exposed to lead for the first time, while the other 17 workers had a history of occupational exposure to this xenobiotic. Workers were subjected to high doses of lead because they did not adhere to the occupational safety and health precautions and did not properly use personal protective equipment.

The second group included 52 male workers chronically exposed to lead for a maximum of 38 years, aged from 23 to 55 years. Workers in that group worked in lead–zinc works as smelters, fitters, and production masters.

The third group was a control group, which included 22 male administrative employees aged from 26 to 59 years. Their blood lead levels were below 10 μg/dl. None of the subjects in this group was occupationally exposed to lead.

Exclusion criteria included history of any chronic diseases (such as immune disorders, diabetes, arterial hypertension, coronary artery disease, and malignant neoplasm) and abnormal physical examination findings, especially symptoms and signs of any infectious diseases.

The experimental setup has been approved by the Bioethics Committee of the Medical University of Silesia in Katowice No. KNW/0022/KB1/108/14.

### Laboratory procedures

#### Blood collection

Blood was drawn once from workers chronically exposed to lead and from subjects in the control group. However, blood was drawn twice from workers subchronically exposed to lead, at the beginning of the study and after a period of subchronic exposure to lead.

Blood samples obtained from each subject were taken from the cubital vein using vacuum tubes (Vacuette^®^; Greiner-Bio, Frickenhausen, Germany) that contained K3EDTA to obtain whole blood or using plain tubes to obtain serum. Blood samples were frozen and stored at −20 °C until used.

#### Determination of lead concentration

PbB assessments were performed by graphite furnace atomic absorption spectrometry using ICE 3400 instrument (Thermo Fisher Scientific Waltham, MA, USA). The laboratory met the requirements of proficiency tests (Lead and Multielement Proficiency—CDC in Atlanta). ClinCal^®^ Whole Blood Calibrator and ClinCal^®^ Serum Calibrator (Recipe, Germany) were used for calibration of the instrument and control materials. ClinCheck Whole Blood Control Levels I, II, and III, and ClinCheck Serum Control Levels I and II were used for quality control. Data were expressed as µg/dl.

#### Determination of zinc protoporphyrin concentration

The blood concentration of zinc protoporphyrin (ZPP) was measured directly using Aviv Biomedical hematofluorometer model 206, using an excitation wavelength of 415 nm and an emission wavelength of 596 nm. The instrument measures the ratio of ZPP fluorescence to the sample (hemoglobin) absorption, displayed as μg ZPP per g of hemoglobin (μg/g Hb). The concentration of hemoglobin in 10 % hemolysate was determined by the cyanmethemoglobin method using Drabkin’s reagent.

#### Determination of uric acid, bilirubin, and albumin concentrations

The serum concentrations of uric acid, albumin, and bilirubin were measured using PerkinElmer biochemical analyzer according to the manufacturer’s instructions. Uric acid and bilirubin concentrations were expressed as mg/dl, while those for albumin were expressed as g/l.

#### Determination of total thiols

The concentration of thiol groups in serum was determined as described by Koster et al. (1986) using 5,5′-dithiobis-(2-nitrobenzoic acid) (DTNB), which undergoes reduction by compounds containing sulfhydryl groups, yielding the yellow anion derivative, 5-thio-2-nitrobenzoate, which absorbs light at a wavelength of 412 nm. This assay was carried out using an automated analyzer PerkinElmer. The results were expressed as μmol per g of protein (P).

#### Determination of total antioxidant capacity (TAC)

Total antioxidant capacity was measured in serum according to Erel (2004). In this colorimetric method, radicals are generated and the antioxidant ability of seminal plasma reduces radical formation. The change in color of ABTS ions (2,2′-azinobis(3-ethylbenzothiazoline-6-sulfonate) is measured as the change in absorbance at 660 nm. This assay was conducted in an automated PerkinElmer analyzer calibrated with Trolox. Data were expressed as mmol/l.

#### Determination of total oxidant status (TOS)

Total oxidant status was measured in serum according to Erel (2005). The assay is based on the oxidation of ferrous ions to ferric ions in the presence of various oxidizing agents in acidic medium. The color change of xylenol orange by ferric ions is measured as the change in absorbance at 560 nm. This assay was conducted in an automated analyzer (PerkinElmer) calibrated with hydrogen peroxide. Data were expressed as µmol/l.

#### Oxidative stress index (OSI)

The percentage ratio of TOS to TAC was used as an OSI.

#### Determination of chemokines

The levels of interleukin-8 (IL-8), eotaxin, interferon gamma-induced protein-10 (IP-10), MCP-1, macrophage inflammatory protein-1β (MIP-1β), and RANTES were determined in serum using a Bio-Plex 200 System (Bio-Rad Laboratories Inc., USA).

The Bio-Plex system is based on three core elements. The first core element is a technology that uses fluorescent magnetic microspheres (beads), each with a distinct color code to permit discrimination of individual tests within a multiplex suspension and allow the simultaneous detection of diverse analyte molecules in each single well of a 96-well microplate. Moreover, the magnetic beads allow for magnetic separation during the washing steps. The second core element is a specific flow cytometer with two lasers and associated optics to measure different molecules bound to the surface of the beads. The third core element is a high-speed digital signal processor that efficiently analyzes the fluorescent output.

The principle of these bead-based assays is similar to that of capture-sandwich immunoassays. The samples and standards were incubated with the antibody-coupled beads (antibodies directed against the desired cytokines were covalently coupled to internally dyed beads) in 96-well plates and washed. Next, the biotinylated detection antibodies specific for different cytokine epitopes were added. After incubation and washing, streptavidin (phycoerythrin solution) was added to bind to the biotinylated detection antibodies on the beads. The suspensions of the washed beads were analyzed using the Bio-Plex System. The software presented the data as both median fluorescence intensity and concentration (pg/ml).

#### Statistical analysis

The statistical analysis was performed using Statistica 9.1 PL software program. Data were expressed as mean ± standard deviation (SD) for normally distributed data and in terms of median and interquartile range (IQR) for non-normally distributed data. Shapiro–Wilk’s test was used to verify normality, and Levene’s test was used to verify the homogeneity of variances. Statistical comparisons were made using *t* test, *t* test with separate variance estimates, Mann–Whitney *U* test, or Chi-squared test. Dependent variables were analyzed using Student’s *t* test and Wilcoxon’s test.

To compare the values of the measured cytokines in workers subchronically exposed to lead and those chronically exposed to lead, the levels of each cytokine in both groups were normalized. Values of cytokine levels measured in workers after subchronic exposure to lead were normalized to the median of the values obtained before exposure, while values of cytokine levels measured in chronically lead-exposed workers were normalized to the median of the values obtained from the control group.

Additionally, regression analysis was performed. The results of each parameter are not normally distributed. In order to obtain normal distributions, the values were log-transformed. However, we obtained a distribution, which enabled us to use multiple regression analyses only for IP-10, MIP-1β, and IL-8. In multiple regression, we analyzed the effects of blood lead level, BMI, age, and smoking on IP-10, MIP-1β, and IL-8 concentrations. Since cytokines concentrations differed significantly between the subchronically exposed group and the chronically exposed group, the group encoding the variable was introduced into the regression equation. A *p* value < 0.05 was considered to be significant.

## Results

Epidemiologic data and blood lead concentrations are presented in Tables 1 and 2.

**Table 1:** Epidemiologic data and lead exposure markers in the group of workers subchronically exposed to lead (*n* = 34)

	Mean	SD
Age (years)	40	13
Exposure duration (days)	40	3
Weight (kg)	79.3	12.5
Height (cm)	176	6.71
Smokers (%)	68 %	–
PbB _before exposure to lead_ (µg/dl)	10.7	7.83
PbB _after exposure to lead_ (µg/dl)	48.7	14.2
ZPP (μg/g Hb)	2.66	0.64

*PbB* blood lead level; *ZPP* zinc protoporphyrin level in the blood

**Table 2:** Epidemiologic data and lead exposure markers in the group of workers chronically exposed to lead and in the control group

	Chronic lead-exposed group *n* = 52		Control group *n* = 22		
	Mean	SD	Mean	SD	*p*
Age (years)	39	8	41	8	0.224
Years of work	13	10	17	9	0.128
Height (cm)	177	5.58	179	8.03	0.112
Weight (kg)	84.2	14.1	88.9	12.2	0.176
Smokers (%)	37 %	–	32 %	–	0.702*
PbB (µg/dl)	36.6	8.60	2.22	1.42	<0.001
ZPP (μg/g Hb)	4.24	1.82	2.38	0.61	<0.001

*PbB* blood lead level; *ZPP* zinc protoporphyrin level in the blood; *p* value–*t* test, p* value–Chi-squared test

The levels of uric acid and bilirubin were significantly higher after a subchronic exposure to lead compared to the baseline by 22 and 35 %, respectively. Similarly, the values of TAC, TOS, and OSI increased by 15, 50, and 33 %, respectively. At the same time, the levels of thiol group and albumin decreased by 5 and 8 %, respectively (Table 3). The levels of IL-8 and MIP-1β were significantly higher after a subchronic exposure to lead compared to the baseline by 34 and 20 %, respectively, while the levels of eotaxin, IP-10, MCP-1, and RANTES did not change after a subchronic exposure to lead (Table 4).

**Table 3:** Levels of uric acid, thiol groups, albumin, and bilirubin and values of total antioxidant capacity (TAC), total antioxidant status (TOS), and oxidative stress index (OSI) before and after subchronic exposure to lead, *p* value–*t* test for dependent variables, p* value–Wilcoxon’s test

	Before exposure		After exposure		Relative change (%)	*p* value
	Mean	SD	Mean	SD		
Uric acid (mg/dl)	6.23	1.00	7.57	1.88	22	<0.001
Thiol groups (µmol/g P)	3.89	0.45	3.68	0.64	−5	0.022
Albumin (g/l)	6.62	0.93	6.07	1.05	−8	0.013
Bilirubin (mg/dl)	0.45	0.24	0.60	0.51	35	0.027*
TAC (mmol/l)	0.76	0.10	0.87	0.14	15	<0.001
TOS (µmol/l)	9.99	7.20	14.9	10.6	50	0.008*
OSI (%)	1.35	0.98	1.79	1.32	33	0.022*

**Table 4:** Levels of interleukin-8 (IL-8), eotaxin, interferon gamma-induced protein-10 (IP-10), monocyte chemoattractant protein-1 (MCP-1), macrophage inflammatory protein-1β (MIP-1β), and RANTES (regulated on activation, normal T cell expressed and secreted) before and after subchronic exposure to lead showed as a median and interquartile range (IQR), *p* value–Wilcoxon’s test

	Before exposure		After exposure		Relative change (%)	*p* value
	Median	IQR	Median	IQR		
IL-8 (pg/ml)	4.30	3.53	5.77	6.37	34	0.047
Eotaxin (pg/ml)	93.0	87.1	106	66.9	14	0.437
IP-10 (pg/ml)	751	713	1052	795	40	0.074
MCP-1 (pg/ml)	33.5	29.9	27.7	38.7	−17	0.248
MIP-1β (pg/ml)	51.7	30.8	62.1	43.7	20	0.002
RANTES (pg/ml)	21,431	3305	20,434	3309	−5	0.174

The level of IL-8 was significantly higher in the group of workers chronically exposed to lead than in the control group by 40 %, while the level of IP-10 was significantly lower by 28 %. The levels of the remaining chemokines did not differ between both groups (Table 5).

**Table 5:** Levels of interleukin-8 (IL-8), eotaxin, interferon gamma-induced protein-10 (IP-10), monocyte chemoattractant protein-1 (MCP-1), macrophage inflammatory protein-1β (MIP-1β), and RANTES (regulated on activation, normal T cell expressed and secreted) in the group of workers chronically exposed to lead and in the control group showed as a median and interquartile range (IQR), *p* value–Mann–Whitney *U* test

	Control group		Chronic lead-exposed group		Relative change (%)	*p* value
	Median	IQR	Median	IQR		
IL-8 (pg/ml)	2.11	0.50	2.95	0.90	40	<0.001
Eotaxin (pg/ml)	95.3	54.6	119	76.7	25	0.083
IP-10 (pg/ml)	1237	676	887	552	−28	0.036
MCP-1 (pg/ml)	14.6	9.01	15.3	14.2	5	0.178
MIP-1β (pg/ml)	65.4	29.8	58.2	28.2	−11	0.197
RANTES (pg/ml)	21,607	1576	20,752	2059	−4	0.109

Multiple regression analysis showed that the duration of lead exposure (subchronic vs. chronic) affects IP-10 and MIP-1β concentrations. Besides, the BMI, age, and smoking habits did not significantly affect the levels of those cytokines. In consistence with these results, comparisons made between the medians of the normalized cytokine levels in subchronically and chronically lead-exposed groups showed significant differences in the normalized values of IP-10, MCP-1, and MIP-1β levels between the two groups (Table 6).

**Table 6:** Normalized cytokine levels obtained from subchronically and chronically lead-exposed groups showed as a median and interquartile range (IQR), *p* value–Mann–Whitney *U* test

	Subchronic exposure *N* = 34		Chronic exposure *N* = 52		*p* value
	Median^a^	IQR	Median^b^	IQR	
IL-8 (%)	134	80–229	140	114–157	0.980
Eotaxin (%)	114	66–138	125	87–168	0.180
IP-10 (%)	140	83–189	72	55–100	<0.001
MCP-1 (%)	83	32–148	105	79–176	0.007
MIP-1β (%)	120	96–181	89	70–114	<0.001
RANTES (%)	95	95–101	96	91–101	0.660

^a^Median of the values of cytokine levels measured in workers after subchronic exposure to lead that were normalized to the median of the values obtained before exposure

^b^Median of the values of cytokine levels measured in chronically lead-exposed workers that were normalized to the median of the values obtained from the control group

## Discussion

Results of the present study showed that subchronic exposure to lead is able to increase uric acid level in the blood. However, increased uric acid level may be not only due to the subclinical renal function impairment but also due to altered purine metabolism. In our previous study, we showed a positive association between lead exposure and the activity of xanthine oxidase, which is responsible for uric acid formation from purines being degraded (Kasperczyk et al. 2013). In consistence with these results, positive correlations between blood lead levels and uric acid concentrations were observed by Hernández-Serrato et al. (2006) in subjects environmentally exposed to high doses of lead and by Wang et al. (2002) and Ehrlich et al. (1998) in battery factory workers. Besides, Khan et al. (2008) reported an increased uric acid level in a group of chronically lead-exposed workers (PbB = 29.1 μg/dl). Similarly, a study on workers chronically exposed to high doses of lead reported same results (PbB = 80.9 μg/dl). However, there are human studies showing no association between lead exposure and uric acid levels (Konishi et al. 1994; Roels et al. 1994).

Uric acid acts as a scavenger of ROS and serves as a main antioxidant in human plasma (Gersch et al. 2008). Therefore, the elevation of uric acid level may compensate for the simultaneous decrease in thiol group level. As a result, the TAC value was paradoxically increased due to subchronic lead exposure. However, uric acid has also been shown to act as a pro-oxidant. Uric acid is able to react directly with nitric oxide (NO) to form 6-aminouracil. This irreversible reaction, resulting in depletion of NO, can be partially blocked by glutathione (GSH). Therefore, under oxidative stress conditions, when the GSH pool is depleted, uric acid may induce endothelial dysfunction (Gersch et al. 2008; Xie et al. 2015). It is well documented that lead exposure induces GSH depletion (Kasperczyk et al. 2014). In light of this, the role of uric acid in the defense against lead toxicity can be divergent and may depend on the specific redox conditions of the particular microenvironment. What makes this issue more complicated is that uric acid also exerts pro-inflammatory properties (Hayden and Tyagi 2004).

In in vitro studies, both lead and uric acid have been reported to induce the expression of IL-8 via MAPKs and NF-κB signaling pathways (Lin et al. 2015; Liang et al. 2015). IL-8 is secreted by multiple cell types in response to pro-inflammatory stimuli and serves as a strong chemotactic agent for neutrophils. Neutrophils may cause oxidative damage to tissues. Consequently, IL-8 may be involved in many inflammatory diseases, such as rheumatoid arthritis, gouty arthritis, asthma, and acute respiratory distress syndrome. Besides, experimental studies showed that IL-8 plays a role in promotion of angiogenesis and metastasis (Lin et al. 2015; Yan et al. 2015). Since this study showed elevated level of IL-8 due to both subchronic and chronic lead exposure, then IL-8 may also display negative effects in humans exposed to lead toxicity.

Similarly, MCP-1 expression and level have been shown to be increased by lead and uric acid through MAPKs and NF-κB signaling pathways in in vitro and experimental studies (Kanellis et al. 2003; Kumawat et al. 2014; Liang et al. 2015; Soliman et al. 2015; Zhou et al. 2012). In addition, uric acid has been reported to increase the expression and levels of RANTES in vitro (Zhou et al. 2012). MCP-1 is released due to pro-inflammatory stimuli and displays a chemoattractive activity on monocytes, basophils, and lymphocytes, thus plays a role in the allergic reactions (Luster and Rothenberg 1997). RANTES also belongs to the chemokine family and is responsible for recruiting a variety of leukocytes into the inflammation sites, such as lymphocytes, macrophages, eosinophils, and basophils (Aldinucci and Colombatti 2014). Levels of both MCP-1 and RANTES were not significantly affected by subchronic and chronic exposure to lead. However, a comparison made between the normalized values of MCP-1 in subchronically and chronically lead-exposed workers showed that the influence of lead on MCP-1 level might be opposite depending on the exposure period.

As in the case of MCP-1 and RANTES, the present study did not confirm the influence of lead exposure on eotaxin level. Eotaxin acts as a chemokine-stimulating eosinophils chemotaxis. Besides, eotaxin induces the release of eosinophils from the bone marrow, their aggregation, and their respiratory burst activity (Rankin et al. 2000).

Results of the present study indicated that a subchronic exposure to lead, apart from increasing the level of IL-8, might induce inflammation via increasing the level of MIP-1β, a member of the MIP-1 family, which orchestrates acute and chronic inflammatory host responses by recruiting pro-inflammatory cells, especially lymphocytes and monocytes (Maurer and von Stebut 2004). Chronic exposure to lead did not significantly affect the level of MIP-1β but influenced the level of IP-10, which serves as a chemokine as well. The level of IP-10 was significantly lower in chronically lead-exposed workers than in the control group. The secretion of IP-10 by lymphocytes depends on IFN-γ level and is related to the Th1-mediated immune response (Antonelli et al. 2014). Therefore, a decrease in IP-10 level due to chronic lead exposure may be caused by lead-induced skewing toward the Th2-mediated immune response as postulated in some experimental studies (Heo et al. 2007; 1996; Hsiao et al. 2011). Besides, it has been postulated that uric acid is also able to trigger the Th2-mediated immune response (Moon et al. 2010). Additionally, multiple regression analysis and comparisons made between the normalized values of IP-10 and MIP-1β in subchronically and chronically lead-exposed workers confirmed that the influence of lead on their levels is divergent in those two different types of exposures. Iavicoli et al. (2006) showed that the effect of lead exposure on cytokine levels in mice depends on the blood lead level. However, results of the present study indicate that lead may affect cytokine levels in different ways depending on the exposure duration rather than blood lead level.

In our previous study on chronically lead-exposed workers, we reported not only a higher level of uric acid but also a higher bilirubin level. In addition, the present study showed an increased level of bilirubin due to subchronic exposure to lead. Bilirubin is the end product of heme degradation. Heme is converted by heme oxygenase (HO) to biliverdin, which is in turn reduced to bilirubin by biliverdin reductase. Bilirubin has been shown to have a strong antioxidant potential against peroxyl radicals; however, it could also exert toxic effects when present in excess (Fuhua et al. 2012; Annabi Berrahal et al. 2007). Several animal studies on rats showed elevated bilirubin levels as a result of lead exposure (Abdel-Moneim et al. 2011; Annabi Berrahal et al. 2007; Ibrahim et al. 2011). Such elevation of bilirubin level may be beneficial owing to its antioxidant properties. In accordance with this, Noriega et al. (2003) showed that bilirubin administration to rats increased GSH level, enhanced the activity of antioxidant enzymes, and decreased the toxicity induced by δ-aminolevulinic acid (ALA). ALA accumulates because of the lead-induced inhibition of δ-aminolevulinic acid dehydratase (ALAD) (Wang et al. 2015). Therefore, high bilirubin level may contribute to the elevation of the TAC value. On the other hand, results of human studies on the role of bilirubin in lead toxicity are not as conclusive as those of the experimental studies. Al-Neamy et al. (2001) and Khan et al. (2008) did not report any significant association between bilirubin level and chronic lead exposure in male workers. The negative results of these studies may be due to the complexity of the possible interactions between lead and heme metabolism. On the one hand, it is well documented that lead inhibits heme biosynthesis (Dobrakowski et al. 2014). Consequently, the depletion of the heme pool may result in its decreased degradation and less bilirubin synthesis. On the other hand, lead may induce heme degradation via induction of the inducible isoform of heme oxygenase (HO-1) (Vargas et al. 2003). Lead may also increase heme degradation via induction of eryptosis (Aguilar-Dorado et al. 2014).

In contrast to uric acid and bilirubin levels, the level of albumin significantly decreased after a subchronic exposure to lead compared to the baseline. Similarly, in our previous study, we reported lower albumin level in chronically lead-exposed workers when compared to the control group. In consistence with our results, Khan et al. (2008) reported decreased serum albumin and total protein levels in lead-exposed industrial workers. Koo et al. (1994) reported decreased albumin mRNA level in rat liver due to lead nitrate administration. This experimental study further supports the results of the human studies.

Albumin is the most abundant plasma protein, which serves to buffer the blood, maintain the osmotic pressure, and as a carrier of many compounds (Guo et al. 2014). Because of their ROS scavenging activity, thiol groups of cysteine residues of albumin determine the plasma redox status (Dobrakowski et al. 2014). Therefore, decreased level of thiol groups, observed in the present study may be secondary to the decrease in albumin level. The second possible explanation for the reduced thiol group level is the well-documented high affinity of lead toward thiol groups (Dobrakowski et al. 2014). In accordance with the human studies, decreased level of thiol groups was also reported in experimental studies in rats (El-Missiry 2000; Tandon et al. 2002). Our previous study on chronically lead-exposed workers also showed decreased thiol group level (Dobrakowski et al. 2014). Interactions between lead and thiol groups may also influence the immune response because it has been proposed that lead may affect lymphocyte functions due to its high affinity for the sulfhydryl groups on T-lymphocyte surface receptors. As a result, antigen processing from monocytes to T lymphocytes may be impaired (García-Lestón et al. 2012).

The development of oxidative stress in chronic lead exposure is well established (Kasperczyk et al. 2014). The decrease in thiol group level and the elevation of TOS and OSI values observed in the present study confirm that a subchronic exposure to lead is also able to induce oxidative stress despite increased TAC value.

The results of this study need to be evaluated within the context of its limitations. A major limitation was a limited groups’ size. Besides, the possible confounding role of other pollutants was not taken into consideration.

## Conclusions

Similar to chronic lead exposure, subchronic exposure to lead modifies the blood levels of uric acid, thiol groups, bilirubin, and albumin in humans. Modifications of uric acid and bilirubin levels may be related to the non-enzymatic antioxidant defense mechanisms against lead toxicity and result in increased TAC value. However, this elevation seems to be insufficient to protect against lipid peroxidation and to compensate for thiol depletion. In both subchronic and chronic lead exposures, oxidative stress may be enhanced by the pro-inflammatory action of chemokines, especially IL-8. Besides, lead may affect IP-10, MCP-1, and MIP-1β levels in different ways depending on the exposure duration rather than blood lead level. These results indicate a need to investigate the mechanisms of human adaptation to lead toxicity related to the exposure period.
